# Application of objective structured clinical examination (OSCE) for the evaluation of Kampo medicine training

**DOI:** 10.1186/s12909-022-03264-3

**Published:** 2022-03-25

**Authors:** Marie Amitani, Haruka Amitani, Hajime Suzuki, Suguru Kawazu, Kimiko Mizuma, Kojiro Yamaguchi, Toshimichi Oki, Hideaki Nitta, Takuro Sonoda, Keiko Kawano, Yasuhiro Tanaka, Nanami Uto, Rie Ibusuki, Ryutaro Arita, Shin Takayama, Tadamichi Mitsuma, Toshiro Takezaki, Akihiro Asakawa, Tetsuhiro Owaki

**Affiliations:** 1 Education Center for Doctors in Remote Islands and Rural Areas, Kagoshima University Graduate School of Medical and Dental Science, Kagoshima, Japan; 2 Division of Psychosomatic Internal Medicine, Kagoshima University Graduate School of Medical and Dental Sciences, Kagoshima, Japan; 3 Division of Oral and Maxillofacial Surgery, Kagoshima University Graduate School of Medical and Dental Sciences, Kagoshima, Japan; 4 Division of Physiology, School of Medicine, Showa University, Tokyo, Japan; 5 Division of Reproductive Health Nursing, School of Health Science, Faculty of Medicine, Kagoshima University, Kagoshima, Japan; 6 Nagashima-Cho National Health Insurance Dental Clinic, Kagoshima, Japan; 7 Pharmacological Department of Herbal Medicine, Kagoshima University Graduate School of Medical and Dental Science, Kagoshima, Japan; 8 Department of Education and Support for Regional Medicine, Department of Kampo Medicine, Tohoku University Hospital, Sendai, Japan; 9 Department of Kampo and Integrative Medicine, Tohoku University Graduate School of Medicine, Sendai, Japan; 10 Department of Kampo Medicine, Aizu Medical Center, Fukushima Medical University, Fukushima, Japan

**Keywords:** Kampo medicine, OSCE,, Kampo education

## Abstract

**Background:**

The purpose of this study was to develop an objective, content-valid, and reliable assessment method for Kampo medicine using an objective structured clinical examination (OSCE) for the assessment of clinical competence in Kampo medicine.

**Methods:**

We developed a blueprint followed by a list of 47 assessment items and three task scenarios related to clinical competence in Kampo medicine. An eight-member test committee checked the relevance of the assessment items on a Likert scale. We calculated a content validity index and content validity ratio, and used the Angoff method to set the passing threshold. We trained a total of nine simulated patients with three assigned to each scenario. We conducted an OSCE for 11 candidates with varying medical abilities, and conducted three stations per person, which were evaluated by one evaluator in one room by direct observation. We used video recordings to test the inter-rater reliability of the three raters. We used the test results to verify the reliability of the assessment chart.

**Results:**

The inter-rater reliability (intraclass correlation coefficient [2,1]) was 0.973. The reliability of the assessment chart for each scenario (Cronbach’s α) was 0.86, 0.89, and 0.85 for Scenarios 1, 2, and 3, respectively. The reliability of the assessment chart for the whole OSCE (Cronbach’s α) was 0.90.

**Conclusions:**

We developed a content-valid new OSCE assessment method for Kampo medicine and obtained high inter-rater and test reliabilities. Our findings suggest that this is one of the most reliable evaluation methods for assessing clinical competence in Kampo medicine.

**Supplementary Information:**

The online version contains supplementary material available at 10.1186/s12909-022-03264-3.

## Background

In recent years, medical education has become increasingly internationalized [1]. For education in Kampo medicine, a type of ancient traditional Japanese medicine that has been handed down based on the apprenticeship system, the World Federation for Medical Education has listed “interface with complementary medicine” as an internationally accredited item [2].

In Japan, the Ministry of Education, Culture, Sports, Science and Technology has included Kampo medicine in its Model Core Curriculum for Medical Education [3], and Kampo has been incorporated into the medical education curriculum of 80 universities nationwide. In addition, the Kampo curriculum has been incorporated into the Model Core Curriculum for Dental Education in Japan [4]. This has led to calls for the introduction of Kampo education into not only medical school, but also dental school curricula.

**Fig. 1:**
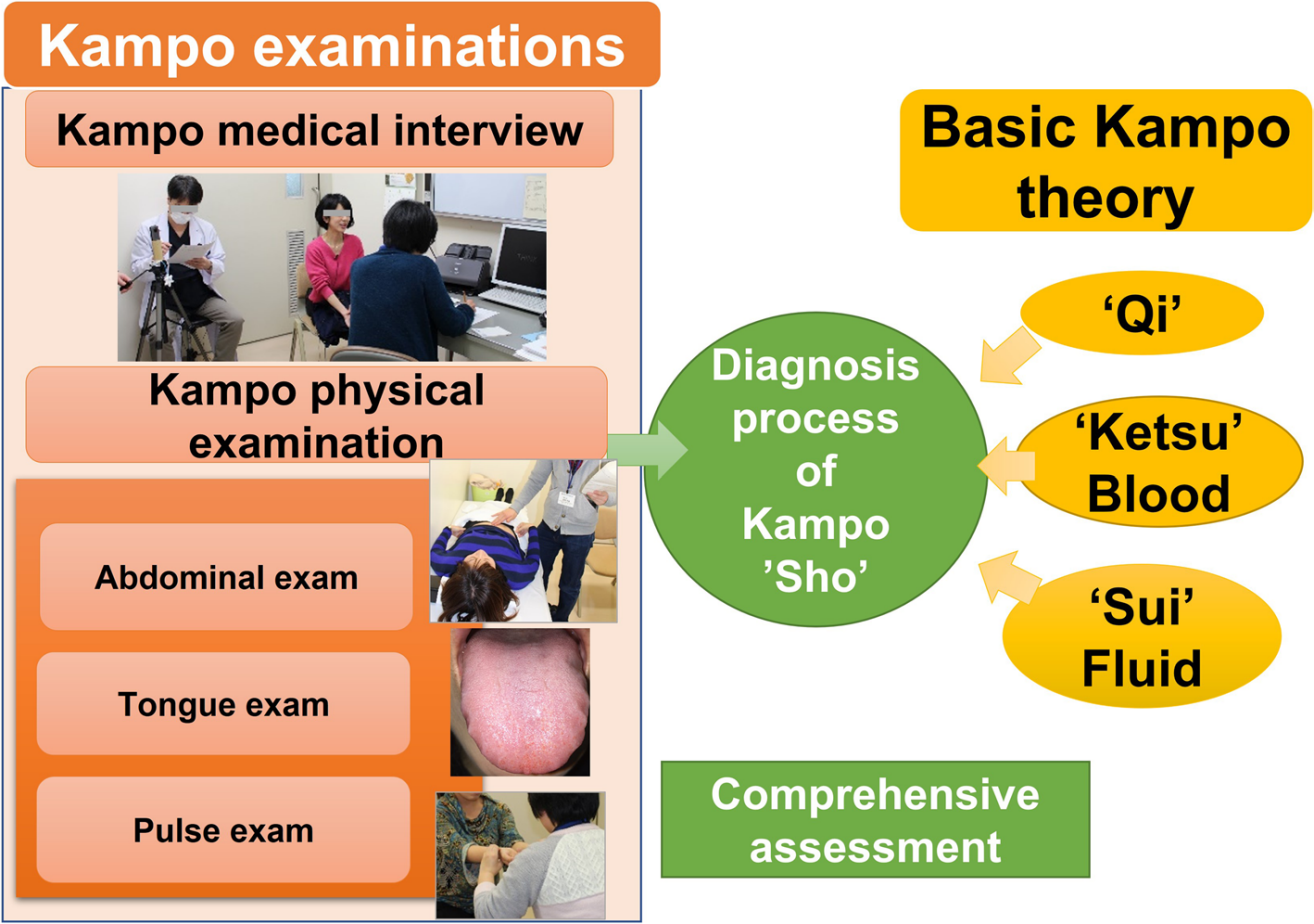
Diagnostic process of Kampo medicine, called “Sho”. Kampo clinical examinations consist of a medical interview and a Kampo physical examination including abdominal, tongue, and pulse examinations

The diagnostic process of Kampo medicine is unique and differs from that of Western medicine. In the diagnostic process of Kampo medicine, a “Sho” is determined based on a comprehensive assessment that involves characteristic questioning and tongue, abdominal, and pulse examinations, followed by the selection of a Kampo formula based on Kampo medicine theory (see Fig. 1). In the traditional apprenticeship system, learners aspiring to become Kampo specialists learn by studying directly under a limited number of Kampo specialists, and evaluation is at the discretion of each instructor. In the current situation, where there is a shortage of instructors who can teach Kampo, the implementation of Kampo education has been left to the discretion of universities and training institutions, and only a limited number of learners have had the opportunity to learn the clinical skills of Kampo. Most physicians without formal Kampo education have tended to learn Kampo medicine through self-learning and to use Kampo based on Western biomedical diagnoses and theories rather than on traditional Kampo theory [5]. The main reason for this is the lack of a standardized Kampo education system, including the evaluation of medical techniques, in Japan [6].

Based on the results of a survey conducted to understand the educational status of Kampo medicine in the medical schools of 80 universities in Japan [6], Kampo medicine education is typically assessed by means of examinations, reports, and book reports. Although the need for Kampo medicine education is increasing and various universities have proposed such initiatives, to our knowledge, no studies have been conducted on how to evaluate proficiency in the clinical techniques of Kampo medicine, which is usually required. Therefore, the Kampo medicine education system has long been based on an apprenticeship system that is passed down from a limited number of skilled users of Kampo medicine to their disciples. However, international demand for the expansion of Kampo education in current medical education is increasing. Therefore, it is necessary to standardize Kampo medicine within the framework of medical education and to promote its spread internationally.

Simulation tests are known to be effective in the field of medical education. A simulation test with a simulated patient is a performance-based test in which the simulated patient plays the role of a patient according to a predetermined scenario [7]. Such a test is highly effective as a method of assessing clinical competence and as such, has been used in objective structured clinical examinations (OSCEs) [8].

Our hypothesis was that an objective evaluation of clinical competence could be introduced to Kampo medicine, a traditional complementary medicine. Our research questions were, first, whether it is possible to transition Kampo medicine education from an apprenticeship system to a standardized evaluation system, and second, whether it is possible to develop an OSCE that can evaluate Kampo medicine competency with high reliability and content validity. Therefore, the purpose of this study was to develop an objective, content-valid, and reliable assessment method for Kampo medicine using an OSCE for the assessment of clinical competence in Kampo medicine.

## Methods

### Development of an objective structured clinical examination (OSCE)

A blueprint was created by positing a wide range of competencies that would presumably be necessary for learners. This study developed an OSCE focusing on the competence of clinical skills within the competency of Kampo medicine. The competencies for Kampo education were based on the competencies proposed by the Japan Council for Kampo Medical Education, which consists of Kampo educators from 82 medical schools [9]. The assessment criteria were set by an OSCE in response to the blueprint (Supplementary Material 1). A literature survey regarding Kampo medicine was conducted, and a 50-item assessment chart was created after extracting high-frequency Kampo medicine findings. A test committee (*n* = 8: six doctors and two dentists; mean ± standard deviation experience working in Kampo medicine physician: 15.1 ± 5.7 years) responded to a rating sheet on a Likert-type scale regarding the relevance of each item, from 1 = questionable to 4 = essential.

A content validity index (CVI) was calculated based on the percentage of total items rated by the experts as either 3 or 4. A CVI of 0.8 or higher was considered valid and indicated the representativeness and clarity of the checklist items [10]. Of the 50 assessment items, those with a CVI lower than 0.8 were excluded. Three of the 50 items had a CVI of less than 0.8. In the study, 47 items were included in the assessment chart.

**Fig. 2:**
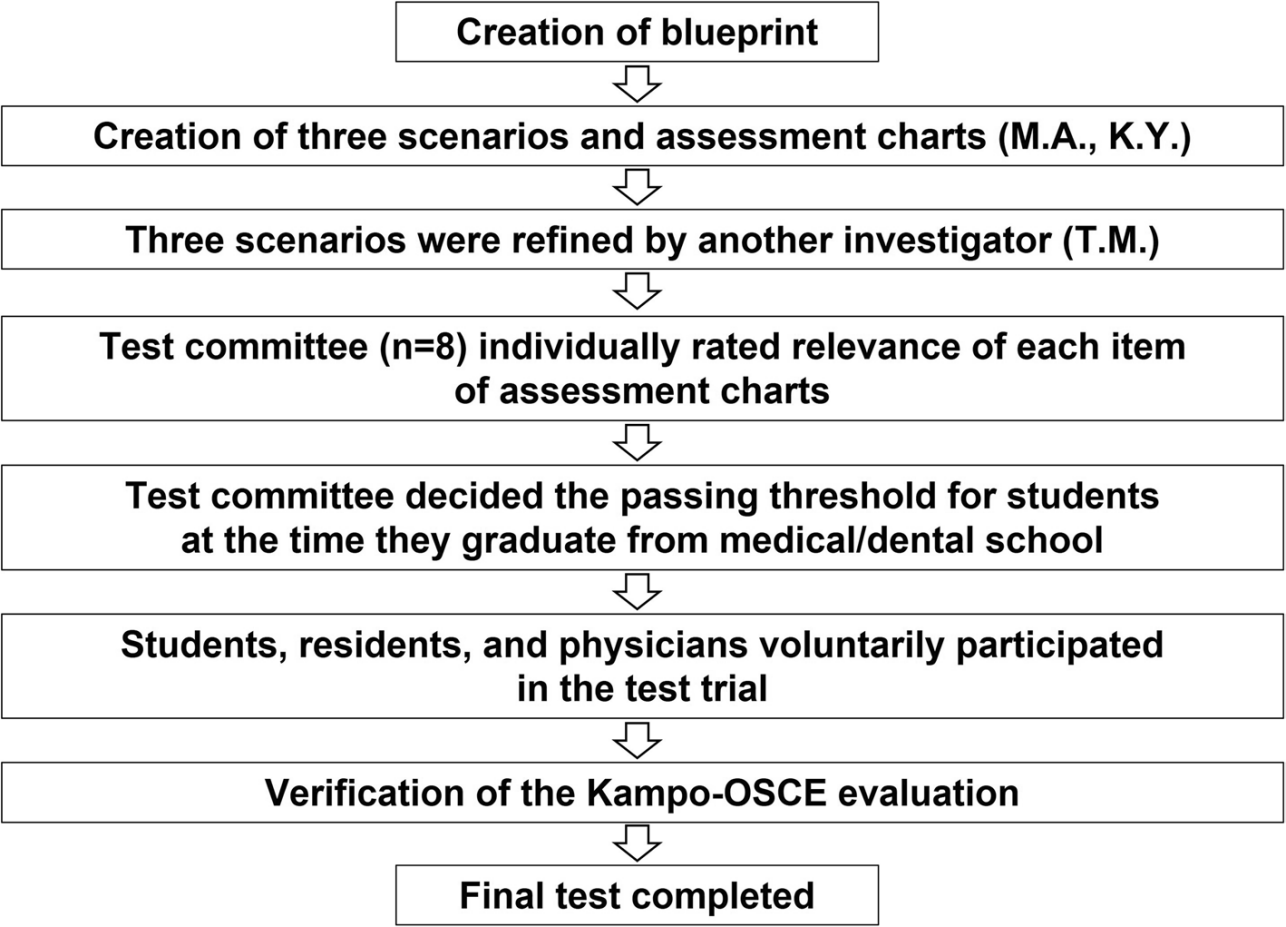
Flowchart of the development of the objective structured clinical examination (OSCE)

Then, in accordance with the blueprint, three problem scenarios corresponding to the Kampo-based concepts of “qi”, “blood”, and “water” were established (Supplementary Material 2). The assessment charts for the three scenarios with the same level of difficulty (Supplementary Material 3) were developed by the investigators (M.A. and K.Y.) and verified for content validity by a Kampo specialist (T.M.). Each of the investigators had over 10 years of experience working in Kampo medicine. A flowchart detailing the development of the Kampo-OSCE is shown in Fig. 2.

### Training of simulated patients according to the scenarios

Fifteen applications for simulated patients were obtained as a result of a public advertisement. Findings for the simulated patients based on a Kampo-based examination were obtained by two investigators (M.A. and K.Y.). Nine of the 15 simulated patients consistent with the Kampo medicine findings of the scenario were selected. These nine simulated patients were then divided into three groups based on similarities in their Kampo-based questionnaires and medical examination findings. Next, the scenarios were distributed to the simulated patients, and guidance involving two explanation sessions (6 h in total) was carried out in advance. The two investigators confirmed that all simulated patients in the same groups could perform according to the scenario, and that the performances among the simulated patients were consistent.

### Training highly reliable raters

Problem scenarios and assessment charts were distributed in advance to six raters, who each had over 8 years of experience working in Kampo medicine. All raters were provided with an explanation of the evaluation method and practiced the evaluation methods.

### Passing threshold

We used Angoff methodology to calculate the passing threshold. The test committee took part in a two-session Angoff standard-setting procedure. In the first session, the judges individually used their professional judgment to estimate the score that a minimally competent final-year student would get on each tested element of the Kampo-OSCE. In the second session, all test committee members worked toward consensus [11].

### Participants

The study participants were recruited through voluntary applications for the Kampo learning program. The participants were medical students, dental students, residents, physicians, and dentists from grades 1 to 5 (Supplementary Material 4). The total number of participants was 11. Three of the 11 participants had more than 8 years of experience working in Kampo medicine. This study was approved by the ethical review board of Kagoshima University (IRB No: 180345–640). Written informed consent was obtained from all participants.

### Conducting the Kampo-OSCE trial

The Kampo-OSCE was administered to participants attending the Kampo learning program. Each examinee was assigned three test problems with approximately the same level of difficulty (Supplementary Material 5). The simulated patients played the role of a patient according to the scenario, and the examinee conducted an interview and a Kampo-oriented physical examination, including tongue, abdominal, and pulse examinations, on the simulated patient. One rater evaluated the patient according to the assessment chart by direct observation. This data will be denoted as “Data 1”. Each test consisted of a 20-min clinical examination of Kampo medicine findings. The examinee wrote the information on the obtained findings on a descriptive sheet. The descriptive information was then used for post-test feedback. To verify whether the evaluators’ assessments and so forth were being carried out appropriately, video recordings were made with the consent of the participants.

### Reliability and validity of the Kampo-OSCE evaluation

To evaluate inter-rater reliability, three raters evaluated one examiner’s video while viewing the recorded video in separate rooms without exchanging information with each other. Six videos were evaluated. These data were denoted as “Data 2”. Using Data 2, the intraclass correlation coefficient (ICC) was calculated and used as an indicator of inter-rater reliability. Next, a two-way random model was employed for the ICC (2,1).

The reliability of the assessment chart for each scenario was evaluated based on the internal consistency of the 47 questions in each scenario was evaluated using Cronbach’s alpha for Data 1. The reliability of the assessment chart for the whole OSCE was assessed by showing the internal consistency of summing the total score of each of the three scenarios using Cronbach's alpha for Data 1.

To evaluate validity, we compared between the group with little experience and the group with Kampo clinical experience.

### Statistical analysis

SPSS (v. 23.0; IBM Corp., Armonk, NY, USA) was used for all statistical analyses. Inter-rater reliability was calculated using data from six examiners’ videos of three raters, with two-way random model ICC (2,1). Cronbach’s alpha was calculated as an indicator of the internal consistency of the 47 items in each scenario and that internal consistency of the three total scenario points. The criteria of Cronbach’s α and the ICC were 0.00–0.20 = slight, 0.21–0.40 = fair, 0.41–0.60 = moderate, 0.61–0.80 = substantial, 0.81–1.00 = almost perfect [12]. The results are expressed as the mean values ± standard error (S.E.). Comparisons between the two groups were performed using an unpaired Welch’s t test between two groups.

## Results

### Inter-rater reliability

The inter-rater reliability obtained from the evaluation using video recordings by the three raters was ICC (2,1) = 0.973 (95% asymptotic confidence interval [asympt. CI]: 0.900–0.996) (Table 1).

**Table 1:** Inter-rater reliability (3 evaluators * 6 examinee videos)

variable	ICC (2, 1)	95% asympt. CI lower	95% asympt. CI upper
Scenario Total Points	0.973	0.900	0.996

ICC (2, 1): Intra-class correlation coefficients with two-way random model. 95% asympt. CI: 95% asymptotic confidence interval of ICC (2, 1)

Scenario Total Points: Total score for each scenario (total score of 47 items)

### Reliability of the assessment chart for each scenario

The reliability of the assessment chart for each scenario is shown in Table 2. High reliability was found for each of the three problem scenarios (Cronbach’s α = 0.86 [95% asympt. CI: 0.79–0.91], 0.89 [95% asympt. CI: 0.83–0.93], and 0.85 [95% asympt. CI: 0.78–0.91] for Scenarios 1, 2, and 3, respectively).

**Table 2:** Reliability of the evaluation scale for each scenario

	Scenario 1	Scenario 2	Scenario 3
Cronbach’s α (95% asympt. CI)	0.86 (0.79–0.91)	0.89 (0.83–0.93)	0.85 (0.78–0.91)

95% asympt. CI: 95% asymptotic confidence interval. Cronbach's α was calculated based on the data from the 11 examinees who took the tests to assess the internal consistency of the 47 items for each of the three scenario tests

### Reliability of the assessment chart for the whole OSCE

The reliability of the assessment chart for the whole OSCE was high (Cronbach’s α = 0.90 [95% asympt. CI: 0.75–0.90]) (Table 3).

**Table 3:** Reliability of the evaluation scale for the whole OSCE

	whole OSCE (three scenario)
Cronbach’s α (95% asympt. CI)	0.90 (0.75–0.90)

95% asympt. CI: 95% asymptotic confidence interval. Cronbach's α was calculated based on the data from the 11 examinees who took the tests to assess the internal consistency of the three scenario total scores

### Comparison between nonexperts and experts for the three test scores

**Fig. 3:**
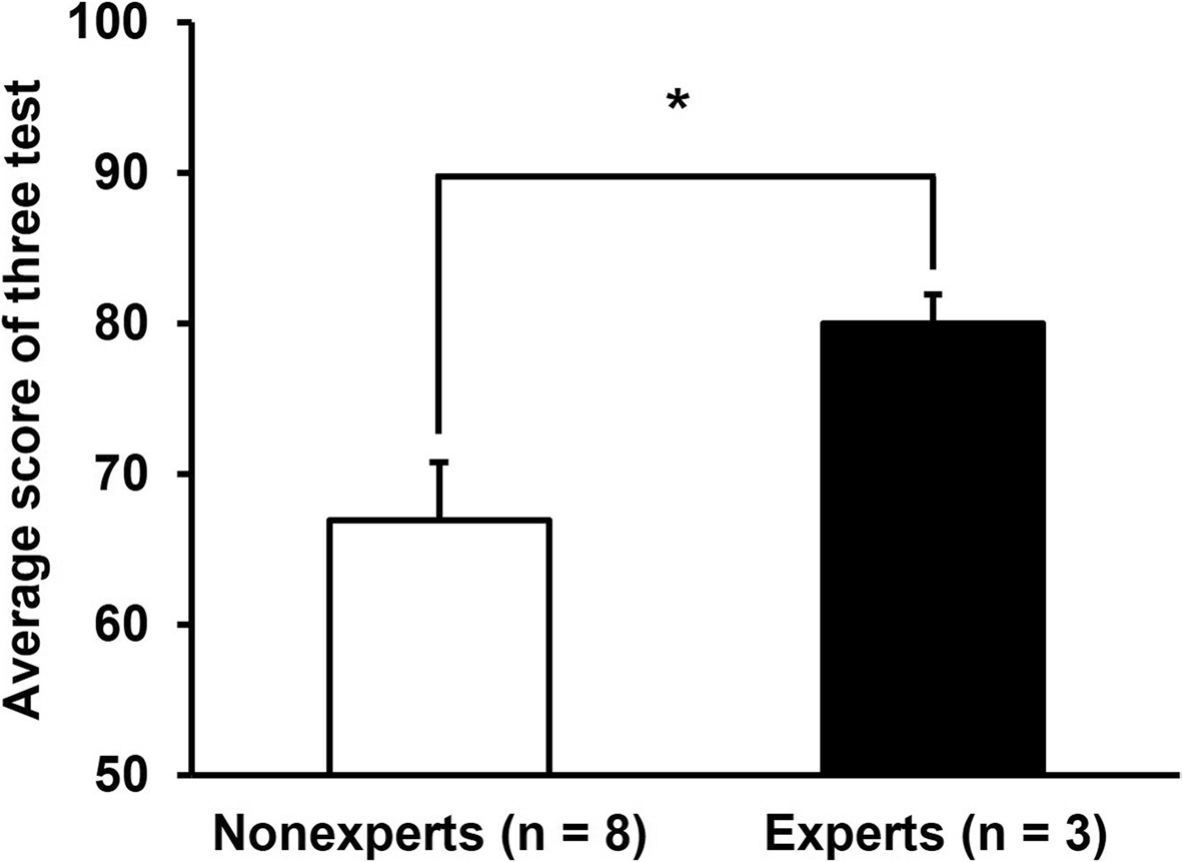
Experts who were examinees with more than 8 years of experience scored higher than nonexperts (experts [*n* = 3], 80.0 ± 1.9; nonexperts [*n* = 8], 66.9 ± 3.8)

Examinees with more than 8 years of experience are described as experts in the text. The results of a comparison of average scores on the three tests between experts and other examinees (nonexperts) indicated that the experts scored significantly higher (experts [*n* = 3], 80.0 ± 1.9; nonexperts [*n* = 8], 66.9 ± 3.8) (Fig. 3).

### Passing threshold

**Fig. 4:**
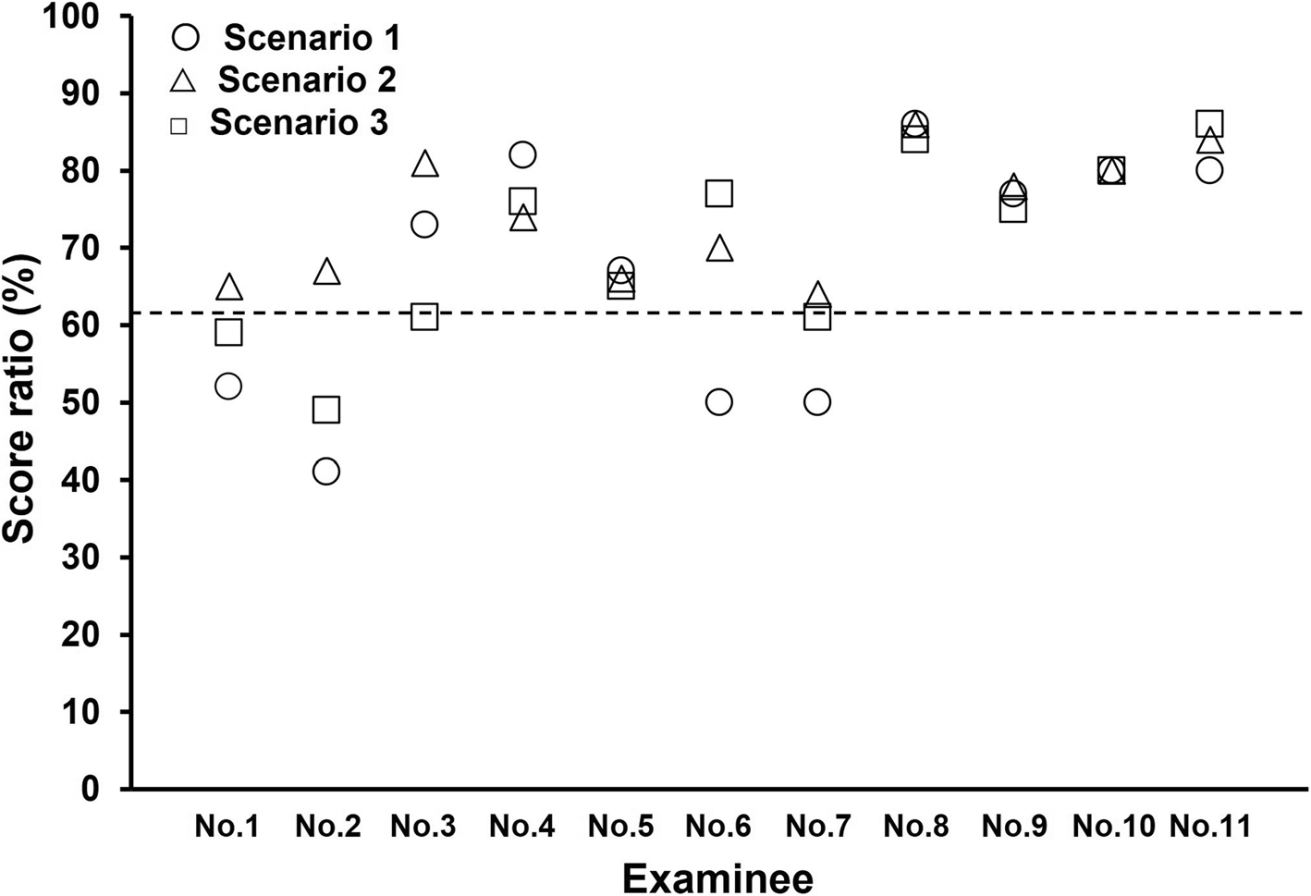
Score ratio for each examinee (*n* = 11) across the three tests. The score ratio was calculated by dividing the number of correct answers by the total number of questions. The dotted line indicates the 62% passing threshold calculated using the Angoff method

Figure 4 shows the score ratio for each examinee (*n* = 11) who took each of the three scenario tests and the passing threshold. The score ratio was calculated by dividing the number of correct answers by the total number of questions. According to the calculation using Angoff methodology, the test committee selected a passing threshold of 62%.

## Discussion

Our research questions were whether it is possible to shift the education of Kampo medicine from an apprenticeship system to a standardized evaluation system, and whether it is possible to develop an OSCE that can evaluate competence in Kampo medicine with high reliability and content validity.

OSCEs with simulated patients are reported to be more effective than role-play education among students [13]. In this study, we developed a new OSCE assessment method involving simulated patients for Kampo medicine education. In addition, since OSCEs have been reported to be an appropriate means of assessing communication skills in medical education in regard to the assessment of medical interviews and attitudes [14], the OSCE in this study included interviews, Kampo-based medical examinations, and patient attitudes as assessment items. An important implication was that we have succeeded in introducing a reliable and valid OSCE into Kampo medicine education, which suggests that Kampo medicine education could transition from an apprenticeship system to a standardized evaluation system within the framework of medical education. Regarding whether this OSCE meets the needs of Kampo, the clearly defined assessment items were assumed to have enabled the assessment of standardized clinical skills in Kampo medicine. This allowed us to develop clinical skills and Kampo assessment items that had only been conducted by a limited number of Kampo instructors, as an evidence-based assessment standard. Further study is needed to determine whether the Kampo-OSCE developed in this study meets existing needs.

### Number of stations and content validity

In this study, we developed a highly reliable evaluation method focusing on Kampo medicine techniques, which is one of the consensus competencies among Kampo educators in Japanese medical schools, as well as a blueprint of the diagnostic process and basic theory of Kampo. Based on this blueprint, we selected a Kampo formula for a selection task and created three task scenarios.

We set three tasks and conducted the Kampo-OSCE in three stations. Regarding whether this number of stations was sufficient, it has been reported that eight stations is a reasonable compromise as a screening test in terms of high sensitivity (88–89%) and specificity (83–86%) [15]. To our knowledge, no reports of OSCEs for Kampo examinations have been published, so it is unclear how many stations are needed for evaluating Kampo. However, the findings of the present study suggest that the reliability of the test with regard to the number of stations may be maintained, even if the number of tasks for the evaluation of experienced candidates is small, while three or more tasks are desirable for the evaluation of inexperienced candidates.

In our study, Cronbach’s α between stations 1, 2, and 3 showed a reliability of 0.59–0.95. One candidate had a low α, suggesting that the reliability may not be maintained across the three tasks for candidates with little experience in Kampo medicine. However, the three tasks were highly reliable for candidates with experience in Kampo. This suggests that for inexperienced examinees, differences between stations may be seen when they are affected by nervousness or not skilled at a task. On the other hand, examinees with lengthy experience in Kampo were less affected by the content and circumstances of the task and demonstrated a certain level of Kampo examination ability.

### Reliability

The reliability of the Kampo-OSCE developed in this study was sufficiently high. A previous systematic review regarding the inter-rater reliability for communication skills assessment noted that the agreement between reviewers was 0.45 [16].

In the present study, we established one evaluator for each station and conducted the evaluations under direct observation. Afterwards, three evaluators conducted individual evaluations while watching video recordings, and the inter-rater reliability of the three evaluators was examined. A high degree of reliability was obtained between the three raters, so it could be said that the raters could evaluate the Kampo consultations similarly.

On the other hand, direct visual and video-recorded assessments differ. The inter-rater reliability in this study was assessed using video recordings, and the results showed high reliability. Previous reports have found that assessment using clinical imaging and video correlate well with OSCEs [17]. Therefore, because high reliability was obtained in the video-based evaluation in this study, the inter-rater reliability was judged to be high for the Kampo-OSCE. It will be necessary to consider whether it is better to evaluate the OSCE under direct visual assessment or to evaluate it after the fact by video recording.

### Limitations

There are several limitations to this study. The first is the number of cases. The number of cases in this study does not cover all the important clinical concepts of Kampo medicine. The blueprint shows scenarios regarding important clinical concepts from the perspective of Kampo medicine, including qi stagnation, qi counter flow pattern, static blood, and fluid retention, but not qi deficiency and blood deficiency. Therefore, it may be desirable to add more stations for qi deficiency and blood deficiency.

The second is the training of simulated patients. In this study, two evaluators with sufficient experience in Kampo medicine conducted a preliminary examination to improve the content validity of the simulated patients, and confirmed that the scenario and simulated patients were consistent in their findings. However, the more experienced examinees felt uncomfortable with the differences between the real patients and scenarios. This has been pointed out previously in a study in which 28 internists took part in an OSCE of a cardiac physical examination using three methods: real patients, cardiac audio–video simulations associated with “normal” standardized patients, and a cardiac patient simulator [18]. The correlation coefficients between participants’ physical examination skills and diagnostic accuracy were 0.39 (*P* < 0.05) for real patients, 0.29 for standardized patients, and 0.30 for the cardiac patient simulator, and were significantly higher for real than for standardized patients and the audio–video system combination [18], which suggests that the diagnostic accuracy is higher when using real patients; this may be a limitation of using simulated patients for physical examination evaluations. Since the test was conducted on simulated patients, the experienced doctors were more likely to notice the differences between the findings of real and simulated patients, which was confusing. The challenge is to collect simulated patients that match the scenario. In addition, specialists may feel uncomfortable with the simulated patients, which may result in not getting a high score. It is necessary to increase the number of medical specialists who take the test and examine the reliability of the test.

Third, it will be necessary to train evaluators in order to apply the test as a standardized test for students in the future. Whether reliability can be maintained when the number of evaluators increases is an issue for the future.

## Conclusion

In this study, we developed a new OSCE assessment method for clinical competence in Kampo medicine, and confirmed its high inter-rater and test reliability. Our results suggest that it is one of the most reliable assessment methods for assessing clinical competence in Kampo medicine, which is expected to become increasingly important in the future as the introduction of Kampo medicine education progresses. Establishing an objective assessment method will be a step toward developing Japanese traditional medicine as a part of an international education.

## Supplementary Information

 **Additional file 1.** Competency,Blueprint and Basic terms of Kampo medicine.

**Additional file 2.** Scenario for simulated patients.

**Additional file 3.** Assessmentchart.

**Additional file 4.** Characteristics of the participants.

**Additional file 5.** Kampo-OSCE time schedule.

## Data Availability

The datasets used and/or analyzed in this study are available from the corresponding author by request.

## Abbreviations

CI: Confidence interval
CVI: Content validity index
ICC: Intraclass correlation coefficient
OSCE: Objective structured clinical examinations
