# Supplementary material for: Application of objective structured clinical examination (OSCE) for the evaluation of Kampo medicine training
Source: BMC Med Educ. 2022 Mar 25;22:202. doi: 10.1186/s12909-022-03264-3 (PMC8957151; doi:10.1186/s12909-022-03264-3)
Supplement: Supplementary file 1 — Additional file 1. Competency,Blueprint and Basic terms of Kampo medicine. [file 12909_2022_3264_MOESM1_ESM.docx]

**Supplementary Material 1. Competency, Blueprint and Basic terms of Kampo medicine.**

**Competency:**

**1 Overview the concept of ‘SHO’**

**(Yin and Yang, Emptiness and Truth, Cold and Heat, Front and Back, Five Organs, Six Disease Positions)**

**Theoretical concepts of qi, blood, and water**

**2 Outline the kampo diagnosis process ‘Shi-shin’ (inspection (complexion, color, tongue diagnosis), listening, inquiry, and palpation (pulse diagnosis and abdominal diagnosis).**

**Blueprint:**

| **Kampo diagnostic process’Sho’pattern** | | | **General concepts** | | | | | |
| --- | --- | --- | --- | --- | --- | --- | --- | --- |
|  |  |  | **qi** | | | **blood** | | **fruid** |
|  |  |  | **qi deficiency** | **qi stagnation** | **qi counter flow pattern** | **blood deficiency** | **static blood** | **fruid retention** |
| **Shi-shin** | **Inspection** | **Tongue examination** |  | Hangekobokuto (Scenario 2) | Ryokeijutsukanto (Scenario 3) |  | Keishibukuryogan (Scenario1) | Ryokeijutsukanto (Scenario 3) |
|  | **Listening** |  |  |  |  |  |  |  |
|  | **Inquiry** |  |  |  |  |  |  |  |
|  |  |  |  |  |  |  |  |  |
|  |  |  |  |  |  |  |  |  |
|  |  |  |  |  |  |  |  |  |
|  | **Palpation** | **Pulse examination** |  |  |  |  |  |  |
|  |  | Abdominal examination |  |  |  |  |  |  |

Ryokeijutsukanto covered both concepts of qi stagnation and fruid retention.

**Basic terms of Kampo medicine:**

| Shishin (four examination) | **the four examinations consist of inspection, listening, inquiry, and palpation** | |
| --- | --- | --- |
| Inspection | **a method of examination collecting clinical information from a patient visually. This includes tongue examination.** | |
| Listening | **a method of examination collecting clinical information from a patient by hearing and smelling** | |
| Inquiry | **a method of examination asking about a patient's history and complaints** | |
| Palpation | **a method of examination collecting clinical data from a patient by touching and pressing the body surface. This includes pulse and abdominal examination** | |
| Sho 'pattern' | **Diagnosis of a patient's signs and symptoms coprehensively based on theories of Kampo medicine.** | |
| qi, blood, fluid | **Physiological factors in the theory of Kampo medicine.** |  |
