# Supplementary material for: Application of objective structured clinical examination (OSCE) for the evaluation of Kampo medicine training
Source: BMC Med Educ. 2022 Mar 25;22:202. doi: 10.1186/s12909-022-03264-3 (PMC8957151; doi:10.1186/s12909-022-03264-3)
Supplement: Supplementary file 2 — Additional file 2. Scenario for simulated patients. [file 12909_2022_3264_MOESM2_ESM.docx]

**Supplementary Material 2. Scenario for simulated patients.**

**Scenario 1**

[Basic Information]

Name: Patient A

Gender: Female

Age: 40 years old

Height: 158cm

Weight: 58kg

Occupation: Clerk

[Patient information]

I am a 40-year-old housewife, married at 27, with two boys, ages 10 (grade 4) and 8 (grade 2). I am currently working as a clerk. My husband works in the civil engineering field. There have been no major illnesses.

Since I was in my twenties, I have had strong menstrual cramps and headaches before and during my periods. I have seen information programs on TV about the fear of headaches, and I have visited a neurosurgeon and had an MRI done on my head, but no abnormalities have been pointed out. I have also seen a gynecologist, but was told that it was just my constitution and that I should wait and see.

　 Since I turned 40 years old, I have been experiencing stiff shoulders and headaches have been getting stronger. I am rather hot and sweaty, but I also feel cold hands and feet and hot flashes.

I'm confident in my physical strength and I'm not an angry person, but I do get frustrated when I see my children running wild at home.

　 When I talked to my friend, she said, "Isn't Kampo medicine good for improving your constitution? He recommended this hospital to me.

[Facial expression, tone of voice, and demeanor]

Facial expression is calm. The tone of voice is a little louder and more energetic.

[Character]

Rough. Cheerful.

‘How are you doing today?’

I'm here because I have a headache.

I have had headaches since I was in my twenties, and I thought it was my constitution, but when I talked to a friend about it, she recommended Kampo herbal medicine, so I came to see her.

Recently, my shoulders have been getting stiff. I think it's just my age, but I'm hoping it will get better.

[Current medical history]

Complaint: Headache

Nature: A clammy feeling (site: not centered in the back of the head. Whole head)

Degree: Not enough to fall asleep, but enough to want to take painkillers (about 4/10)

Onset: Since I was in my 20s

Persistence: especially bad before menstruation and throughout the day

Progress: I have had headaches since I was in my 20's. In my 40's, I started to feel stiff shoulders, cold hands and feet, and hot flashes on my face. (Not so much hot flashes).

Trigger: Premenstrual

Aggravating factor: menstruation

Relaxant factors: None in particular

Other symptoms: stiff shoulders, cold hands and feet, facial hot flashes, irritability, palpitations

**(In order of priority when asked: stiff shoulders => palpitations => hot flashes => irritation => cold)**

**Whenever someone asks, "What else? Whenever you are asked, answer one at a time. Do not answer more than one at a time.**

When did this happen?

　　　I have been prone to headaches since I was in my twenties.

What kind of headache?

　　　It's a constrictive headache.

When does the headache get stronger (worse)?

　　　"I get a headache before my period. When I'm not menstruating, I don't get headaches as much.

Do headaches get stronger when cold (in cold weather)?

　　　I don't think it matters much.

Is there a relationship between symptoms and weather?

　　　I don't think it matters.

Do you have any other symptoms?

　　　　I'm also very stiff in the shoulders.

Do you have a cold?

　　　　"I'm rather hot. Sometimes my face gets hot.

[Summary of symptoms]

Headache: (+)

Tinnitus: Occasional ringing in both ears.

Dizziness: sometimes wobbly.

Hot (+) face

Sweat: Those who sweat easily by nature

Dry mouth (-) (No thirst. No dry mouth.

Palpitations: Sometimes for 10 minutes. It subsides spontaneously. No abnormality in ECG test.

Bowel movement: Tendency to constipation, about once every 3-4 days (a little hard, but not colicky). When constipated, my lower abdomen feels a little distended.

Abdominal pain: None

Back pain: None

Numbness in the legs: None

Cold hands and feet: A little cold.　No hot water bottles or other warming devices. (Baths are pleasant but not too hot, no long baths, no chills)

Appetite: Good

Sleep: Good

Irritability: Pre-menstrual. When the child is out of control.

Mood: No depression. No need to cry.

Motivation: Good. Active and mobile.

Fatigue (tiredness): None

Menstruation: First menstruation 12 years old. Severe menstrual pain, 28-day cycle, lasting 5 days. Prone to irregularity under stress. Menstrual flow is normal. Two weeks before last menstruation.

Sometimes there are clots of menstrual blood. The sash is white with a little yellow.

**Others If you are asked, please answer "Yes.**

Some veins in my legs are floating.

The complexion is dull.

Bruises from falls are easy to leave behind.

[Medical history]

Childhood illnesses: None

Diseases in adults: None.

Hospitalization history: None

Surgical history: None

Trauma: None

[Family History]

Father: 70 years old. High blood pressure

Mother: 68 years old. Health

I don't have a brother.

[History of medical visits

Neurosurgery: 6 months ago (MRI of my head showed no abnormalities)

Gynecologist: 6 months ago (told to wait and see)

Internal medicine: 1 month ago (no abnormalities in thyroid test)

[Medication]

Over-the-counter medication: painkillers for headaches. Nutritional drinks when tired.

Prescription drugs: None

Allergies, side effects of medications: None

[Health checkup]

Examined annually. No abnormalities.

Blood pressure 130/80 mmHg

[Lifestyle]

Eating habits: Likes sweets and chocolate

Exercise: None in particular

Sleep: 6 hours. Good sleep

Bowel movement: Good. Regular bowel movement once a day.

Smoking: None

Drinking: Occasional drinking

[Psychosocial background]

Education: Junior college graduate with middle grade.

Marriage: Married at age 27. Married for 13 years.

Occupation: Office work (5 days a week, 9:00-16:00)

Hobbies: Talking with friends, enjoying PTA activities even though they are hard work.

Stress: I am not a person who feels much stress, but my children do not listen to me.

Spouse Information: Age 42. Civil engineering. Health. Angry person. (When my husband gets angry, he basically changes the subject cheerfully. Sometimes he talks back. Doesn't care.

Children: 10 years old (male, 4th grade), 8 years old (male, 2nd grade)

[Interpretive model]

I've been told it's my constitution, and I think it's my age.

Anxiety: None in particular

Preferred tests: None

Desired Treatment: I just want to get better as much as possible.

What is the last thing you want to do? If you ask.

I would be happy if I could get better with Kampo herbal medicine.

| Examination site | Finding | Examinees' behavior | Findings |
| --- | --- | --- | --- |
| Complexion | I'm neither red-faced nor pale. | How are you looking? | It's dull. |
| Lips | Dark red | "What color are your lips? | Dark red. |
| Skin | No drying | He touched his hand, "Is your skin dry? | No dryness. Slightly moist. |
| Nail (e.g. fingernail, toenail) | Not easy to break | She looked at her nails and asked, "What nails? | No split lines, no wisps, no easy cracking. |
| Sweat | Be hot and sweaty | Does it make you sweat? | be hot and sweaty |
| Tongue | Color: Black | What color is your tongue? | Dark red. |
|  | Dimensions |  | No atrophy or enlargement |
|  | Tooth marks: None | Any teeth marks? | There are no teeth marks. |
|  | Moss | How's your tongue? | Thin white moss. |
|  | tongue veins | After examining the lingual veins. | "The lingual veins are distended. |
| Pulse | Depth | I did a pulse check. | (All at once)  Depth is slightly submerged, strength 3, speed 3, pulse width 3, tension 3, slip reluctance 3. |
|  | Strength |  |  |
|  | Speed |  |  |
|  | Pulse width |  |  |
|  | Tension |  |  |
|  | Sullen |  |  |
| Abdoment | Sweating of the abdominal wall | Touch your belly. "Sweating? | A little self-sweat. |
|  | Skin temperature of the abdominal wall | Touch your stomach, "What's the temperature? | No decrease in skin temperature |
|  | Increased intestinal peristalsis | Intestinal peristaltic sounds are | All clear, sir. |
|  | Physical strength | ventral force | 4 |
|  | Tension in the rectus abdominis muscle | Tension in the rectus abdominis muscle is | None (mild) |
|  | Impediment in the heart | impediment in the heart of a person | No, sir. |
|  | Feeling of frustration in the chest | Thoracic dyspepsia is | No, sir. |
|  | Retrograde palpitation | palpitations (of the heart) | No, sir. |
|  | Gastroduodenal consonant | the sound of water striking something | No, sir. |
|  | Feeling aggrieved (at) | those who resort to petty tricks | No, sir. |
|  | Paravalvular resistance and tenderness | The tender point of the blood is | The left side is more tender than the right side, and there is an induration at the position of the second lateral finger below the umbilicus. |
| Foot | Edema: None | Swelling (edema?) | No, sir. |
|  | Cold feet: Yes | Cold feet. | I have coldness in the tips of my hands and feet. |

**Scenario 2**

[Basic Information]

Name: Patient B

Gender: Female

Age: 40 years old

Height: 155cm

Weight: 48kg

Occupation: Housewife

[Patient information.]

I am a 40-year-old housewife, married at 29, with a 9-year-old boy (grade 3). My husband is a civil servant.

There have been no major illnesses.

I have always been a very caring person, and I often held back from saying what I wanted to say. My friends are good company and I like to spend time with them over tea and talking. I have been married for 10 years, but my mother-in-law is very strong-minded and interferes in many things at home. We have many family gatherings, and at New Year's, I had to help out as a daughter-in-law at a gathering of relatives, so I spent a lot of time feeling overwhelmed. My mother-in-law told me that I should have my third-grade boy take an entrance exam, and recommended that he attend a cram school, but I was not willing to do so. My mother-in-law calls me every day to talk about cram schools and lessons. Recently, she often comes to my house to persuade my child to take the entrance exam.

Gradually, when I thought about my mother-in-law, I began to feel pain in my chest and felt as if my heart was being pinched. I couldn't sleep well at night because I was worried that my mother-in-law would say something to me again. I don't feel irritated, but I feel depressed and sigh a lot.

　 My husband, who was worried about me, recommended that I see a doctor, and he recommended this hospital. I visited the internal medicine department, but was told that there were no abnormalities in the X-rays or respiratory function tests.

I don't want to take antidepressants or anti-anxiety drugs because I am afraid of them. I am thinking that I can take Kampo herbal medicine.

[Facial expression, tone of voice, and attitude]

Dark expression, low voice. Sighs a lot.

[Personality]

Mild. Introverted

‘How are you doing today?’

I'm anxious and distressed.

About two months ago, I started to feel a tightness in my chest and I don't know what to do. I can't sleep at night because of anxiety.

My mother-in-law has been coming to my house a lot lately, interfering with my children's entrance examinations and other domestic matters. I was very depressed even before I saw her on New Year's Day, but even after New Year's Day, I feel distressed when I think about my mother-in-law, and I can't sleep for fear that she will say something to me again.

[Current medical history]

Complaint: Chest congestion

Nature: A hazy feeling in the chest

Area: Chest to throat

Degree: Does not interfere with daily life. Degree: Not enough to interfere with daily life, but enough to do the bare minimum of housework.

Onset: About two months ago

Duration: All day long

Progress: Two months ago, I started to feel a tightness in my chest, and about a month ago, I started to have trouble sleeping.

Trigger: When your mother-in-law contacts you.

Aggravating factor: When I am thinking about my mother-in-law

Relaxation factor: when talking with friends

Other symptoms: insomnia, heart palpitations, feeling of being choked, mood swings

**(The order of priority for answering when asked: insomnia => depressed mood => feeling of constriction in the throat => palpitations)**

**Whenever someone asks, "What else? Whenever you are asked, answer one at a time. Do not answer more than one at a time.**

When did this happen?

　　　It's been about two months.

How does the chest get stuck?

　　　My chest is always hazy and uncomfortable.

When are the symptoms more severe?

　　　When my mother-in-law informs me that she is coming to my house, my heart starts to bother me.

Do headaches get stronger when cold (in cold weather)?

　　　I don't think it matters.

Is there a relationship between symptoms and weather?

　　　I don't think it matters.

Do you have any other symptoms?

　　　　I can't sleep at night. I can't sleep at night and my throat feels like it's stuck.

Do you have a cold?

　　　　"I'm neither hot nor cold. More of a cold person.

Are there times when you feel depressed?

　　　　　"I often feel anxious. I wonder what my mother will say.

[Summary of symptoms]

Headache: (-)

Tinnitus: (-)

Dizziness (-)

Hot flashes (-)

Sweat: No sweat

Dry mouth (heavy drinking) (-), Dry mouth (-)

Palpitations: Sometimes for 10 minutes. It subsides spontaneously. No abnormality in ECG test.

Bowel movement: No constipation. Regular bowel movement once a day.

Abdominal pain: None

Back pain: None

Edema: swelling easily

Numbness in the legs: None

Cold hands and feet: None. No use of hot water bottles or other warm appliances. (Baths are nice, but not long ones.)

Appetite: Decreased. No desire to eat.

Sleep: I can't fall asleep. It takes me several hours to fall asleep; I get under the covers at 10pm and don't fall asleep until around 2am. I wake up soon after that.

Frustration: More depressed than frustrated.

Mood: Depressed. Anxious. Not happy. Sometimes tears come out.

Motivation: I do the chores I have to do, but I don't feel motivated to do them.

Fatigue (tiredness): Yes

Menstruation: First menstruation 12 years old. Menstrual pain is not severe. 28-day cycle, lasting 5 days. Menstrual flow is normal. Two weeks before last menstruation.

**Others If you are asked, please answer "Yes.**

Sighs a lot

Prone to stress

burp frequently

My head is heavy and I don't feel refreshed.

[Medical history]

Childhood illnesses: none

Diseases in adults: None.

Hospitalization history: None

Surgical history: None

Trauma: None

[Family History]

Father: 70 years old. High blood pressure

Mother: 68 years old. Health

I don't have a brother.

[History of medical visits]

Respiratory medicine (one month ago) conducted X-ray, EKG, and respiratory function tests, and was told that there were no abnormalities.

Gastroenterologist (1 month ago) performed a gastroscopy and was told there was nothing abnormal.

[Medication]

Over-the-counter medication: None

Prescription drugs: None

Allergies, side effects of medications: None

[Health checkup]

Examined annually. No abnormalities.

Blood pressure 110/60 mmHg

[Lifestyle]

Eating habits: I like Japanese food.

Exercise: None in particular

Sleep: Insomnia. Can't fall asleep. It takes me several hours to fall asleep; I get under the covers at 10pm and don't fall asleep until around 2am. Woke up soon after that. (I don't remember much about my dreams. I don't have scary dreams.

Bowel movement: Good. Regular bowel movement once a day.

Smoking: None

Drinking: No

[Psychosocial background]

Education: Junior college graduate with middle grade.

Marriage: Married at age 29

Occupation: Housewife

Hobbies: Chatting with friends.

Stress: Relationship with mother-in-law

Spouse Information: Age 42. Public servant. Serious (husband does not try to get between her and mother-in-law).

Child: 9 years old (3rd grade) boy

[Interpretive model]

There's nothing wrong with my tests, and I don't know what to do.

Anxiety: I have chest pains, I can't sleep at night, and I feel anxious.

Preferred tests: None

Preferred Treatment: I would like to be cured with Chinese herbal medicine.

What is the last thing you want to do? If you ask.

The other hospital said it was a problem with my feelings, but I'm not sure about tranquilizers, so I'm hoping that Chinese herbal medicine will help.

| Examination site | Finding | Examinees' behavior | Findings |
| --- | --- | --- | --- |
| Complexion | I'm neither red-faced nor pale. | How are you looking? | as you see |
| Facial expression | dark (in colour) | Sighing and looking worried. | as you see |
| Lips | Red | "What color are your lips? | normal flush |
| Skin | No drying | He touched his hand, "Is your skin dry? | There is no drying. |
| Nail (e.g. fingernail, toenail) | Not easy to break | She looked at her nails and asked, "What nails? | No split lines, no wisps, no easy cracking. |
| Sweat | No sweat. | Does it make you sweat? | A simulated patient answers |
| Tongue | Color: Black | What color is your tongue? | normal flush |
|  | Dimensions. | How big is it? | Slightly enlarged. |
|  | Tooth marks: None | Any teeth marks? | There are teeth marks. |
|  | Moss. | How's your tongue? | There is white moss. |
|  | Angry tongue veins:. | After examining the lingual veins. | The lingual veins are not distended. |
| Pulse | Depth | I did a pulse check. | (All at once)  The depth is midway between floating and sinking, strength 2, speed 3, pulse width 3, tension 3, and sliding reluctance 3. |
|  | Strength. |  |  |
|  | Speed. |  |  |
|  | Pulse width |  |  |
|  | Tension. |  |  |
|  | Sullen |  |  |
| Abdoment | Sweating of the abdominal wall | Touch your belly. "Sweating? | No sweat |
|  | Skin temperature of the abdominal wall | Touch your stomach, "What's the temperature? | No decrease in skin temperature |
|  | Gut-wrenching sound | Intestinal peristaltic sounds are | All clear, sir. |
|  | Physical strength | ventral force | 3 |
|  | Tension in the rectus abdominis muscle | Tension in the rectus abdominis muscle is | No, sir. |
|  | Impediment in the heart | impediment in the heart of a person | Yes, sir. |
|  | Feeling of frustration in the chest | Thoracic dyspepsia is | Mild, yes. |
|  | Retrograde palpitation | palpitations (of the heart) | No, sir. |
|  | Gastroduodenal consonant | the sound of water striking something | Yes, sir. |
|  | Feeling aggrieved (at) | those who resort to petty tricks | No, sir. |
|  | Paravalvular resistance and tenderness | The tender point of the blood is | No, sir. |
| Foot | Edema: None | Swelling (edema?) | Mild, yes. |
|  | Cold feet: Yes | Cold feet. | No, sir. |

**Scenario 3**

[Basic Information]

Name: Patient C

Gender: Female

Age: 35 years old

Height: 155cm

Weight: 45kg

Occupation: Part-time (supermarket)

[Patient information]

I am a 35-year-old housewife, married at 27, with a 5-year-old girl. I work part-time at a supermarket. My husband is a company employee (sales). There have been no major illnesses.

I am a worrier by nature and often feel anxious.

I have been experiencing dizziness since I was in high school, and about a year ago I started a part-time job at a supermarket, which kept me busy as I worked while raising my children. About a year ago, I started a part-time job at a supermarket and became very busy working while raising my children. I had to do a lot of standing work at the supermarket, and when I had to unload things, I felt dizzy and had to sit down, which made it difficult to continue working. I went to see a neurosurgeon, but he said that there was nothing wrong with the MRI of my head. The ENT said that I had Meniere's disease, but now I only have occasional dizziness. Palpitations often appear as well.

　I visited this hospital today because I heard that a friend of mine experienced the same symptoms and that Chinese herbal medicine was effective.

[Facial expression, tone of voice, and attitude]

The expression on his face is dark and anxious.

[Personality]

Worried

‘How are you doing today?’

I'm here because I'm wobbly.

I used to get dizzy a lot, but lately I've been doing a lot of standing, sitting and standing, and I've been feeling light-headed a lot.

I've been very busy at work lately, but ever since I started wobbling, I've been sitting down at work a lot, which is annoying me.

[Current medical history]

Chief complaint: Wobbliness

Characteristics: Wobbly head. Occasional spinning (almost never).

Degree: I can't go to work and work a full day.

Onset: Started about 6 months ago.

Duration: All day long.

Progress: 6 months ago, she started to feel lightheaded.

Trigger: When standing up. When you wake up from sleep.

Aggravating factor: when under stress

Relieving factor: subsides after sleep. It gets better when I stay in the same position. The "after sleep" refers to posture, not sleep.

Other symptoms: heart palpitations, ringing in the ears, anxiety, heavy head feeling in the morning

When did this happen?

　　　It's been about six months now.

What kind of dizziness?

　　　"I feel lightheaded and dizzy. Sometimes it feels like I'm spinning.

When are the symptoms more severe?

　　　I feel dizzy when I stand or get up.

Does cold weather (cold weather) make me more dizzy?

　　　I don't think it matters.

Is there a relationship between symptoms and weather?

　　　I don't do well in the rain.

Do you have any other symptoms?

　　　I'm having heart palpitations.

Do you have a cold?

　　　"My feet are cold.

Are there times when you feel depressed?

　　　I feel anxious. I feel light-headed, have heart palpitations, and feel depressed when I think about what will happen next.

[Summary of symptoms]

Headache: Occasional. Heavy feeling

Tinnitus: a low-pitched, gargling sound in both ears

Dizziness (+) (mainly lightheadedness and dizziness)

Hot flashes (-)

Sweat: No sweat

Dry mouth (-) (No thirst. No sensation of dryness in the mouth)

Palpitations: Frequent, about 10 minutes. Subsides spontaneously. No abnormality in ECG test.

Bowel movement: No constipation. Regular bowel movement once a day.

Abdominal pain: None

Back pain: None

Edema: feet tend to swell in the afternoon (less intense swelling)

Numbness in the legs: None

Cold hands and feet: My feet get cold. I sleep with socks on. I like to take a bath, but only to warm up.

Appetite: Not much. I don't want to eat, but I can eat.

Sleep: I can't sleep. I can't sleep when I'm thinking.

Shoulder stiffness: (+)

Irritability: When you feel very lightheaded

Mood: I feel anxious.

Motivation: I do the chores that need to be done, but I often sleep through the night because I feel woozy.

Fatigue: Mild

Menstruation: First menstruation 12 years old. Menstrual pain is not severe. 28-day cycle, lasting 5 days. Menstrual flow is normal. Two weeks before last menstruation.

[Medical history]

Childhood illnesses: none

Diseases in adults: None.

Hospitalization history: None

Surgical history: None

Trauma: None

[Family History]

Father: 70 years old. High blood pressure

Mother: 68 years old. Health

I don't have a brother.

[History of medical visits]

Otolaryngologist (2 months ago): I was told I had Meniere's disease, and was given medication for dizziness (Merislon and Adefos), but it did not improve, and I was told that the best thing to do was to avoid stress.

Cardiologist (one month ago) performed an EKG and said there was nothing abnormal.

[Medication]

Over-the-counter medication: None

Prescription drugs: None

Allergies, side effects of medications: None

[Health checkup]

Examined annually. No abnormalities.

Blood pressure 90/60mmHg

[Lifestyle]

Eating habits: None.

Exercise: None in particular

Sleep: I can't sleep.

Bowel movement: Good. Regular bowel movement once a day.

Smoking: None

Drinking: No

[Psychosocial background]

Education: Junior college graduate with middle grade.

Marriage: Married at age 27

Occupation: Part-time (supermarket)

Hobbies: Talking

Stress: Being busy with both work and childcare. My husband doesn't help with the housework.

Spouse Information: Age 42. Company employee (sales). He doesn't help me with housework when he is busy with work.

Child: 5 years old girl

[Interpretive model]

If only the dizziness would get better, he thinks, everything would be fine.

Anxiety: I'm worried that I'm very dizzy.

Preferred tests: None

Preferred Treatment: I would like to be cured with Kampo medicine.

What is the last thing you want to do?

I have been taking medication for vertigo, but it doesn't help, and I have been hoping that somehow Kampo medicine will help.

| Examination site | Finding | Examinees' behavior | Findings |
| --- | --- | --- | --- |
| Complexion | I'm neither red-faced nor pale. | How are you looking? | as you see |
| Facial expression | uneasy | Expressions. | as you see |
| Lips | Red | "What color are your lips? | normal flush |
| Skin | No drying | He touched his hand, "Is your skin dry? | There is no drying. |
| Nail (e.g. fingernail, toenail) | Not easy to break | She looked at her nails and asked, "What nails? | No split lines, no wisps, no easy cracking. |
| sweat | No sweat | Does it make you sweat? | A simulated patient answers |
| tongue | Color: Black | What color is your tongue? | pale red |
|  | Dimensions | How big is it? | Slightly enlarged. |
|  | Tooth marks on tongue : None | Any teeth marks on tongue ? | There are teeth marks on tongue. |
|  | Moss | How's your tongue? | thin white moss |
|  | Angry tongue veins | After examining the lingual veins. | The lingual veins are not distended. |
| Pulse | Depth | I did a pulse check. | (All at once)  Depth is submerged, strength 2, speed 3, pulse width 2, tension 2, slip reluctance 3. |
|  | Strength. |  |  |
|  | Speed |  |  |
|  | Pulse width |  |  |
|  | Tension |  |  |
|  | Sullen |  |  |
| Abdoment | Sweating of the abdominal wall | Touch your belly. "Sweating? | No sweat |
|  | Skin temperature of the abdominal wall | Touch your stomach, "What's the temperature? | No decrease in skin temperature |
|  | Gut-wrenching sound | Intestinal peristaltic sounds are | All clear, sir. |
|  | Physical strength | ventral force | 2 |
|  | Tension in the rectus abdominis muscle | Tension in the rectus abdominis muscle is | No, sir. |
|  | Impediment in the heart | impediment in the heart of a person | Mild stuffiness in the heart. |
|  | Feeling of frustration in the chest | Thoracic dyspepsia is | No, sir. |
|  | Retrograde palpitation | palpitations (of the heart) | It's quite obvious. |
|  | Gastroduodenal consonant | the sound of water striking something | Yes, sir. |
|  | Feeling aggrieved (at) | those who resort to petty tricks | No, sir. |
|  | Paravalvular resistance and tenderness | The tender point of the blood is | No, sir. |
| Foot | Edema: None | Swelling (edema?) | Mild, yes. |
|  | Cold feet: Yes | Cold feet. | My feet get cold. |
