# Supplementary material for: Application of objective structured clinical examination (OSCE) for the evaluation of Kampo medicine training
Source: BMC Med Educ. 2022 Mar 25;22:202. doi: 10.1186/s12909-022-03264-3 (PMC8957151; doi:10.1186/s12909-022-03264-3)
Supplement: Supplementary file 3 — Additional file 3. Assessmentchart. [file 12909_2022_3264_MOESM3_ESM.docx]

| **Supplementary Material 3. Assessment chart.** | | | | | | |  |  |  |
| --- | --- | --- | --- | --- | --- | --- | --- | --- | --- |
|  |  |  | **Examinee's number:** |  |  |  | |  |  |
|  |  |  | **Evaluator's name:** |  |  |  | |  |  |
|  |  |  |  |  |  |  | |  |  |
| No |  |  |  | **He/she asked** | **He/She didn't ask** |  | | **CVI** |  |
| 1 | **History taking** | Main complaint | Onset | □ | □ |  | | 1 |  |
| 2 |  |  | Progress | □ | □ |  | | 1 |  |
| 3 |  |  | Quality | □ | □ |  | | 1 |  |
| 4 |  |  | Location | □ | □ |  | | 1 |  |
| 5 |  |  | Degree | □ | □ |  | | 1 |  |
| 6 |  |  | Mitigation factor | □ | □ |  | | 1 |  |
| 7 |  |  | Aggravating factor | □ | □ |  | | 1 |  |
| 8 |  | Characteristic Accompanying symptom1,2,3,4 | Accompanying symptom 1 | □ | □ |  | | 1 |  |
| 9 |  |  | Accompanying symptom 2 | □ | □ |  | | 1 |  |
| 10 |  |  | Accompanying symptom 3 | □ | □ |  | | 0.88 |  |
| 11 |  |  | Accompanying symptom 4 | □ | □ |  | | 0.75 |  |
| 12 |  | History taking to determine ‘Sho’ | Sensitive to heat or cold | □ | □ |  | | 1 |  |
| 13 |  |  | Chill | □ | □ |  | | 1 |  |
| 14 |  |  | Influence of weather | □ | □ |  | | 0.88 |  |
| 15 |  |  | Menstruation | □ | □ |  | | 0.88 |  |
| 16 |  |  | Perspiration | □ | □ |  | | 1 |  |
| 17 |  |  | Moods and emotions | □ | □ |  | | 1 |  |
| 18 |  |  | Appetite | □ | □ |  | | 1 |  |
| 19 |  |  | Bowel movement | □ | □ |  | | 1 |  |
| 20 |  |  | Fatigue | □ | □ |  | | 0.88 |  |
| 21 |  |  | Sleep | □ | □ |  | | 1 |  |
|  |  |  |  | **He/She implemented** | **He/She implemented,**  **but inappropriately** | **He/She didn't**  **implemente** | |  |  |
| 22 | **Shi-shin** | Inspection | Complexion | □ | □ | □ | | 1 |  |
| 23 | **(Examinations)** |  | Lips | □ | □ | □ | | 1 |  |
| 24 |  |  | Skin | □ | □ | □ | | 1 |  |
| 25 |  |  | Nail | □ | □ | □ | | 1 |  |
| 26 |  | Tongue inspection | Color | □ | □ | □ | | 1 |  |
| 27 |  |  | Size | □ | □ | □ | | 1 |  |
| 28 |  |  | Tooth mark | □ | □ | □ | | 1 |  |
| 29 |  |  | Tongue coating | □ | □ | □ | | 1 |  |
| 30 |  |  | Varicosis of the lingual veins | □ | □ | □ | | 1 |  |
| 31 |  | Pulse examination | Pulse | □ | □ | □ | | 0.88 |  |
| 32 |  | Abdominal examination | Sweating of the abdominal wall | □ | □ | □ | | 0.63 |  |
| 33 |  |  | Skin temperature of the abdominal wall | □ | □ | □ | | 0.88 |  |
| 34 |  |  | Abdominal strength | □ | □ | □ | | 1 |  |
| 35 |  |  | Abdominal muscle tension | □ | □ | □ | | 0.88 |  |
| 36 |  |  | Epigastric discomfort and resistance | □ | □ | □ | | 1 |  |
| 37 |  |  | Hypochondriac discomfort and distension | □ | □ | □ | | 1 |  |
| 38 |  |  | Brisk pulsation in the supra-umbilical region | □ | □ | □ | | 1 |  |
| 39 |  |  | Splashing sound in epigastric region fluid retension in stomach | □ | □ | □ | | 1 |  |
| 40 |  |  | Weakness of the lower abdominal region | □ | □ | □ | | 1 |  |
| 41 |  |  | Para-umblical tenderness and resistance | □ | □ | □ | | 1 |  |
|  |  |  |  | **He/She implemented** | **He/She implemented,**  **but inappropriately** | **He/She didn't**  **implemente** | |  |  |
| 42 | **Communication** | Appropriate attitude toward patients | | □ | □ | □ | | 1 |  |
| 43 |  | An attitude that encourages talking and asking questions | | □ | □ | □ | | 1 |  |
| 44 |  | Easy to understand questions | | □ | □ | □ | | 1 |  |
| 45 |  | Questions that take into account psychosocial background (mind-body unity) | | □ | □ | □ | | 1 |  |
| 46 |  | Summarize patient information and review contents | | □ | □ | □ | | 0.88 |  |
| 47 |  | Patient-centered examination | | □ | □ | □ | | 1 |  |
| 48 |  | Consideration for the patient's emotions | | □ | □ | □ | | 1 |  |
| 49 |  | Provide clear explanations to patients | | □ | □ | □ | | 1 |  |
| 50 |  | Smooth execution of medical examination | | □ | □ | □ | | 0.63 |  |
|  |  |  | |  |  |  | |  |  |
|  |  |  | **Total (2 points each)：** |  |  |  | |  |  |
