# Supplementary material for: Application of objective structured clinical examination (OSCE) for the evaluation of Kampo medicine training
Source: BMC Med Educ. 2022 Mar 25;22:202. doi: 10.1186/s12909-022-03264-3 (PMC8957151; doi:10.1186/s12909-022-03264-3)
Supplement: Supplementary file 4 — Additional file 4. Characteristics of the participants. [file 12909_2022_3264_MOESM4_ESM.docx]

**Supplementary Material 4. Characteristics of the participants.**

| No. | Gender | Qualification/status |
| --- | --- | --- |
| 1 | Male | First-year medical student |
| 2 | Male | Third-year dental student |
| 3 | Female | Fourth-year dental student |
| 4 | Female | Fifth-year medical student |
| 5 | Male | Dentist |
| 6 | Male | Medical doctor |
| 7 | Male | Resident |
| 8 | Male | Resident |
| 9 | Male | Medical doctor  (8 years working in Kampo medicine) |
| 10 | Male | Dentist  (8 years working in Kampo medicine) |
| 11 | Male | Dentist  (8 years working in Kampo medicine) |
