# Supplementary material for: Application of objective structured clinical examination (OSCE) for the evaluation of Kampo medicine training
Source: BMC Med Educ. 2022 Mar 25;22:202. doi: 10.1186/s12909-022-03264-3 (PMC8957151; doi:10.1186/s12909-022-03264-3)
Supplement: Supplementary file 5 — Additional file 5. Kampo-OSCE time schedule. [file 12909_2022_3264_MOESM5_ESM.docx]

**Supplementary Material 5. Kampo-OSCE time schedule.**

|  | Room 1 | Room 2 | Room 3 | Room 4 | Room 5 | Room 6 |
| --- | --- | --- | --- | --- | --- | --- |
|  | Scenario 1 | Scenario 2 | Scenario 3 | Scenario 1 | Scenario 2 | Scenario 3 |
|  | Evaluator 1 | Evaluator 2 | Evaluator 3 | Evaluator 4 | Evaluator 5 | Evaluator 6 |
| Clinical examination  20 min | E 1, SP 1 <OSCE> | E 2, SP 4 <OSCE> | E 3, SP 7 <OSCE> | E 4, SP 2 <OSCE> | E 5, SP 5 <OSCE> | E 6, SP 8 <OSCE> |
| Descriptive sheet  20 min | <WRS> | <WRS> | <WRS> | <WRS> | <WRS> | <WRS> |
| Clinical examination  20 min | E 3, SP 2 <OSCE> | E 1, SP 5 <OSCE> | E 2, SP 8 <OSCE> | E 6, SP 3 <OSCE> | E 4, SP 6 <OSCE> | E 5, SP 9 <OSCE> |
| Descriptive sheet  20 min | <WRS> | <WRS> | <WRS> | <WRS> | <WRS> | <WRS> |
| Clinical examination  20 min | E 2, SP 3 <OSCE> | E 3, SP 6 <OSCE> | E 1, SP 9 <OSCE> | E 5, SP 1 <OSCE> | E 6, SP 7 <OSCE> | E 4, SP 4 <OSCE> |
| Descriptive sheet  20 min | <WRS> | <WRS> | <WRS> | <WRS> | <WRS> | <WRS> |
| Clinical examination  20 min | E 7, SP 1 <OSCE> | E 8, SP 4 <OSCE> | E 9, SP 7 <OSCE> | E 10, SP 2 <OSCE> | E 11, SP 5 <OSCE> |  |
| Descriptive sheet  20 min | <WRS> | <WRS> | <WRS> | <WRS> | <WRS> |  |
| Clinical examination  20 min | E 9, SP 2 <OSCE> | E 7, SP 5 <OSCE> | E 8, SP 8 <OSCE> |  | E 10, SP 6 <OSCE> | E 11, SP 9 <OSCE> |
| Descriptive sheet  20 min | <WRS> | <WRS> | <WRS> |  | <WRS> | <WRS> |
| Clinical examination  20 min | E 8, SP 3 <OSCE> | E 9, SP 6 <OSCE> | E 7, SP 9 <OSCE> | E 11, SP 1 <OSCE> |  | E 10, SP 4 <OSCE> |
| Descriptive sheet  20 min | <WRS> | <WRS> | <WRS> | <WRS> |  | <WRS> |

Each examinee took part in three test sessions.

E: Examinee, SP: Simulated patient, OSCE: Objective structured clinical examinations, WRS: Written descriptive sheet.
